# Eddy Current-Based Identification and Depth Investigation of Microdefects in Steel Filaments

**DOI:** 10.3390/s24165101

**Published:** 2024-08-06

**Authors:** Kim Sang Tran, Bijan Shirinzadeh, Julian Smith

**Affiliations:** 1 Robotics and Mechatronics Research Laboratory (RMRL), Department of Mechanical and Aerospace Engineering, Monash University, Melbourne, VIC 3800, Australia; kim.tran4@monash.edu; 2 Department of Surgery, School of Clinical Sciences at Monash Health, Monash University, Melbourne, VIC 3800, Australia; julian.smith@monash.edu

**Keywords:** microdefects, eddy current signal, reinforced fibers, depths of defects

## Abstract

In the field of quality control, the critical challenge of analyzing microdefects in steel filament holds significant importance. This is particularly vital, as steel filaments serve as reinforced fibers in the use and applications within various component manufacturing industries. This paper addresses the crucial requirement of identifying and investigating microdefects in steel filaments. Eddy current signals are used for the identification of microdefects, and an in-depth investigation is conducted. The core objective is to establish the relationship between the depth of defects and the signals detected through the eddy current sensing principle. The threshold of the eddy current instrument was set at 10%, corresponding to a created depth of 20 µm, to identify defective specimens. A total of 30 defective samples were analyzed, and the phase angles between the experimental and theoretical results were compared. The depths of defects ranged from 20 to 60 µm, with one sample having a depth exceeding 75 µm. The calculated threshold of 10.18% closely aligns with the set threshold of 10%, with a difference of only 1.77%. The resulting root mean square error (RMSE) was found to be 10.53 degrees, equivalent to 3.49 µm for the difference in depth and phase between measured results and estimated results. This underscores the methodology’s accuracy and its applicability across diverse manufacturing industries.

## 1. Introduction

Steel wire manufacturers are encountering challenges when attempting to implement continuous improvement and operational excellence, particularly in terms of cost savings and increased efficiency. These challenges are compounded by the need to enhance product quality to meet the increasingly stringent demands of end-users [1]. Steel filaments are used as reinforced fibers in the aerospace and automotive industries and many other sectors, including for medical equipment and instruments [2]. Due to their function as reinforcement, the quality of these steel fibers has been improved over time [3]. Consequently, steel cord manufacturers have to continually focus on enhancing process capability and productivity throughout the production process. A typical production system follows a sequence starting with initial design requirements, followed by design review and failure mode and effects analysis (FMEA), which plays a critical role in ensuring manufacturing success [4]. The application of FMEA in the field of non-destructive testing (NDT) is of great importance.

Basically, it is necessary to set the initial condition or setting condition for the methodology and technical perspective. In order to achieve this, failure mode and effect analysis is considered as a critical factor in the very first stage of NDT [5,6,7]. Failure analysis contributes not only to non-destructive testing but also to quality control. Based on the target specifications, special characteristics of the manufacturing process are identified, and failure mode will be analyzed when a process is not functioning correctly. Upon studying such failures, generally defined as defects, and reaction plans are devised to minimize costs and mitigate faults in the manufacturing procedure [8,9]. 

In the forefront of advanced manufacturing, quality control assumes paramount importance in enhancing manufacturing processes and uncovering the root causes of failures during production or research and development activities. Addressing the industry’s needs for cost savings, quality enhancement, and problem solving, a few research projects have focused on investigating the effects of failure analysis [10,11]. Consequently, various failure detection methodologies have been developed to meet these objectives.

The significance of failure analysis cannot be overstated as industries strive for operational excellence. Understanding microdefects in steel filaments is paramount, requiring thorough examination due to the complex interplay between monitored signals and defect depth. This approach is essential for ensuring product quality and reliability, particularly in critical sectors such as aerospace and automotive [12,13,14]. By comprehensively addressing these defects, appropriate measures can be taken to prevent potential failures and maintain high standards in manufacturing. Failure analysis is crucial as industries aim for operational excellence, necessitating a deep understanding of micro surface defects in steel filaments. This endeavor requires precise attention to the connection between monitored signals and defect depth, vital for ensuring product quality and reliability [15,16]. A comprehensive grasp of these defects is essential for effective quality control and preventing potential failures, particularly in critical industries like aerospace and automotive.

Due to the application of metal products, the importance of failure analysis is significant, particularly in the application of steel filament within the manufacturing industry. Failures occurring at the final stages of a product’s shelf-life can have severe consequences [17,18]. This is especially true for failures at the micro level, which may not be easily detected during the initial stages, or in the manipulation of microdefects, such as those examined through scanning electron microscopy and atomic force microscope applications. Therefore, the role of early detection and a comprehensive understanding of microdefects adds significant value to quality control practices and micro/nano manipulation [19,20,21,22,23]

As another type of steel filament, stainless steel wire plays a crucial role in the manufacture of surgical/medical instruments for minimally invasive surgery (MIS) and robotic surgery procedures. High-tensile-strength steel wires are embedded in the jaws of graspers to provide a strong grip and precise control over tissues. This enhanced precision is essential for grasping and manipulating tissues with minimal trauma. Additionally, steel wire can be utilized in cutters to ensure clean and precise incisions, minimizing damage to surrounding tissues. Furthermore, steel wire is integral in suturing devices, allowing for secure and efficient suturing during surgical procedures [24,25].

Although steel products play a central role in manufacturing, the investigation of micro surface defects in steel wire remains largely unexplored. While methods such as vision inspection [26], theoretical analysis like finite element analysis, and software applications have been utilized, their practical effectiveness in detecting subtle defects has been constrained [27,28,29]. Existing studies frequently overlook comprehensive examination of micro-level defects, resulting in a notable gap in understanding the root causes of unexpected product issues.

Micro surface defects have been researched by various approaches. For example, vision inspection, theoretical analysis of surface defects, or even software, such as finite element analysis, have been used to understand the deformation of surface defects and their influence. A piezo was utilized in a transducer to detect surface porosity in metal, which was a very complicated procedure in the real application [30,31]. A vision-based methodology was developed to classify defects in workpieces which were stopped at the classification step, and there was no deep understanding about the defect [32]. Moreover, surface defects were studied and researchers only showed some of the defects under very low speed, and there was no detailed analysis about the accuracy of the detection [33]. 

An eddy current-based system was established to detect surface defects on wire rod; however, the diameter of the target specimen is too big and the experiment was carried out with very low speed [34]. In another study, a crack detection system was studied and monitored with an eddy current sensor, but the system required many sensing devices and various system arrangements to identify unacceptable specimens [35].

Another study focused on steel cord, where the filaments were stranded together, and inspection was conducted under a bundle of filaments. However, this study merely captured the visual inspection of broken wire [36]. Similarly, in another study, surface defects were identified, but the investigation stopped there without any exploration or understanding of the depth or nature of the signal detection [37]. Researchers have developed methodologies to predict failures, such as microdefects. They have utilized various tools, including software, imaging techniques, and sensor systems [38]. 

However, these studies have been limited by the speed of manufacturing and the size of defects, lacking in-depth analysis regarding the origin of unexpected defects. It is essential to prepare samples meticulously and understand failure behaviors thoroughly before conducting image analysis using state-of-the-art technologies. In particular, the relationship between eddy current sensing signals and the shape of microdefects requires further investigation by researchers.

This paper investigates the complex domain of failure analysis, which serves as a cornerstone for industries aiming to reduce costs, enhance quality, and probe into the root causes of issues in both production and research and development activities. With a specific emphasis on the identification and examination of micro surface defects in steel filaments, this paper addresses a critical concern within the manufacturing realm. The paper describes an in-depth investigation of microdefects found on steel filaments and establishes the relationship between the phase angle signal, used to define the depth of monitored signals, and the actual depth of microdefects.

## 2. Methodology

### 2.1. Working Principle

**Figure 1:**
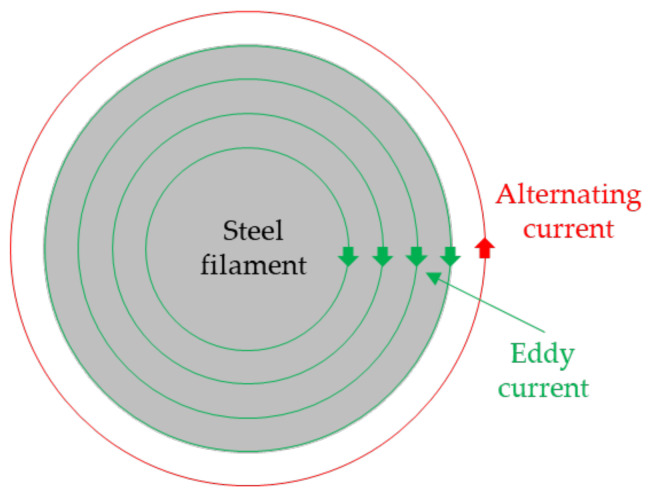
Eddy currents induced around steel filament.

The principle of electromagnetism underpins experiments involving eddy currents [39]. In the filament manufacturing process, a surrounding sensor is employed to detect surface imperfections in the steel filament. The sensor analyzes the eddy current signal upon detecting imperfections in the wire, based on preset conditions. Activation of an input current induces an alternating magnetic field, generating eddy currents within the target object [40], as shown in Figure 1. Utilizing this principle, a sensor coil identifies abnormal signals induced by micro surface defects in the steel wire. The signal amplitude response is then utilized to establish thresholds for detecting desired micro surface defects. 

As outlined in [28], the standard penetration depth (*δ*) is calculated as 19.01 µm, which refers to the point at which the density of eddy currents decreases from 100% at the surface to 37%, where the parameters are shown in Table 1 below:

(1) $$\delta =\sqrt{\frac{2}{\sigma \mu \omega }},$$

To determine the defect’s depth relative to the surface, the ratio between the defect’s depth (z) and the standard penetration depth (δ) is established. This ratio $\left(\frac{z}{\delta }\right)$ is defined as the phase angle in the real part of the current density J(z,t), as described in the formula below [40]:

(2) $$J(z,t)=Real(J{\left(z\right)e}^{j\omega t}=J_{0,max}e^{-\frac{z}{\delta }}\cos\left(\omega t+\alpha_{0}-\frac{z}{\delta }\right),$$

where the depth of the defect and time are denoted by *z* and *t*, respectively. $J_{0,max}$ represents the maximum density at the surface of the target wire, and $\alpha_{0}$ is the phase at t=0 and z=0. Utilizing this principle, the system can detect the desired depth, enabling the establishment of a threshold to identify the depth of the defect.

### 2.2. Experimental Procedure

**Figure 2:**
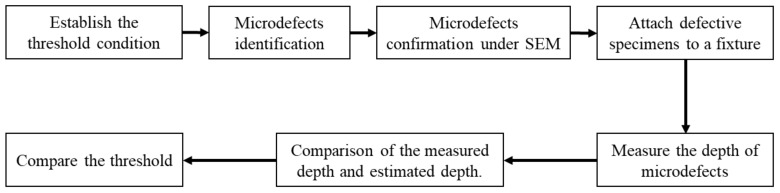
Experimental procedure.

The experiments commenced by establishing the threshold condition, which served to identify the microdefects in the filament. Subsequently, the defective specimens were verified under a scanning electron microscope. In the next step, these unacceptable samples were placed into a mold to secure them before depth measurement. Finally, the measured depth and threshold values were compared with those obtained from estimation, as outlined in Figure 2.

#### Establishing Identification Methods and Defect Analysis

**Figure 3:**
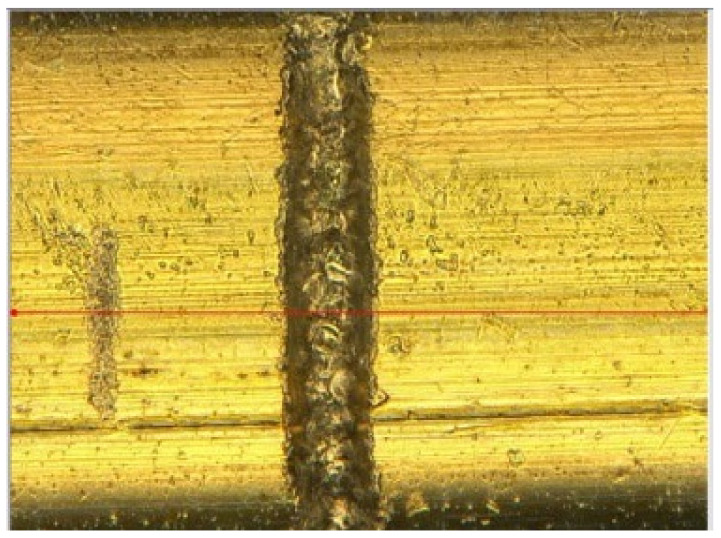
Defect created on steel filament.

In previous research, a crack with a depth of 20 µm was intentionally created on the surface of a sample, which was measured using the reference red line, as illustrated in Figure 3, and the threshold was set at 10%, corresponding to a depth of 20 µm [28]. The experiment was conducted in two batches, with 15 defects selected per batch. Any specimens with imperfections having a depth exceeding 20 µm were removed from the production batch, and the specimens containing these defects were analyzed to validate the set parameters. Additionally, the relationship between the defect depth and the monitored phase angle will be further validated against estimated values.

**Figure 4:**
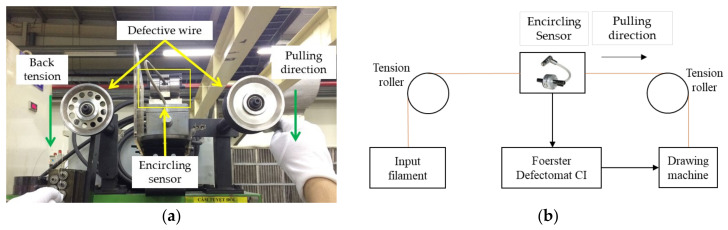
(**a**) Position of encircling sensor; (**b**) block diagram of experiment.

The wire with surface defects was passed through the encircling sensor, allowing the monitor to detect the defect’s position as it passed through the sensor coil. The encircling coil (sensor type 2.865.01-1050) was connected to the instrument (Foerster Defectomat CI, Pittsburgh, PA, USA) which was used to control the experiment process, as depicted in Figure 4.

**Figure 5:**
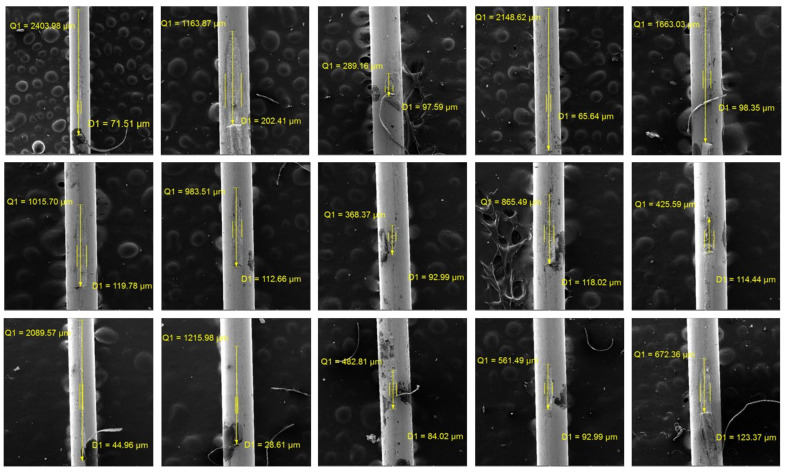
Defects detected under scanning electron microscope.

**Figure 6:**
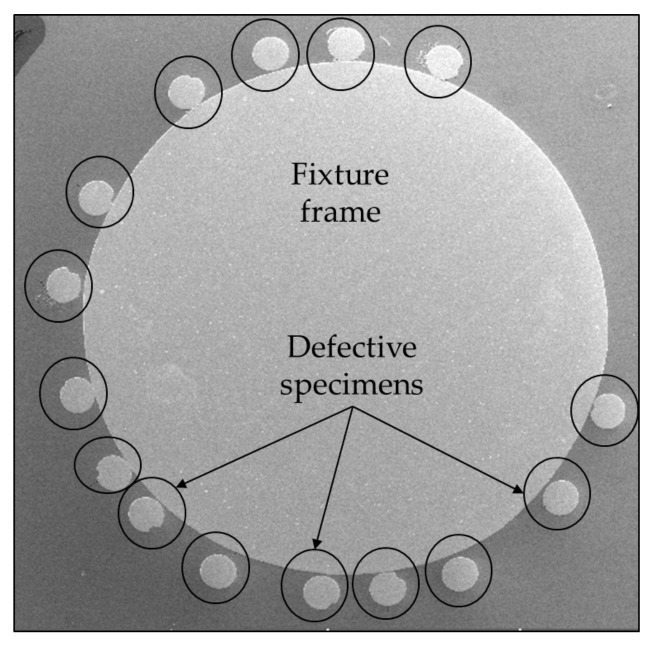
Specimens attached to a fixture frame.

After identifying the defective specimens, they were retracted through the sensor coil to precisely locate the microdefects. The positions of these microdefects on the steel filaments were easily recognized using gloves. Once the locations were identified, they were further narrowed down to verify and confirm their shape using scanning electron microscopy, as shown in Figure 5. Subsequently, these specimens were cut into small pieces, attached to a fixture frame, and placed into a mold to stabilize their position and orientation prior to grinding, as illustrated in Figure 6. The length and width of these defects were then measured using scanning electron microscopy and energy-dispersive spectroscopy.

## 3. Experimental Results

### 3.1. Verification of the Composition inside and outside of Microdefects

**Figure 7:**
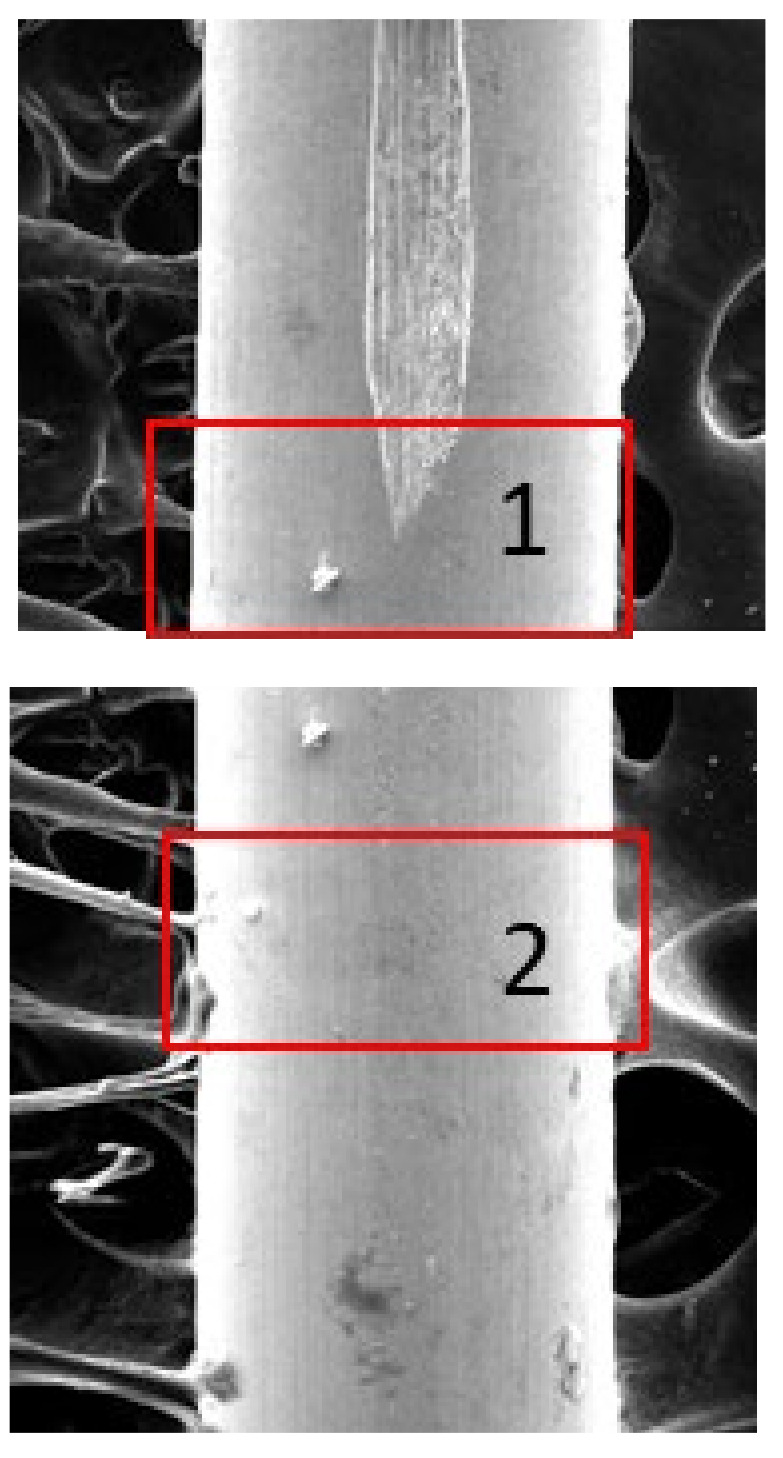
Locations of mapping analysis on a sample.

**Figure 8:**
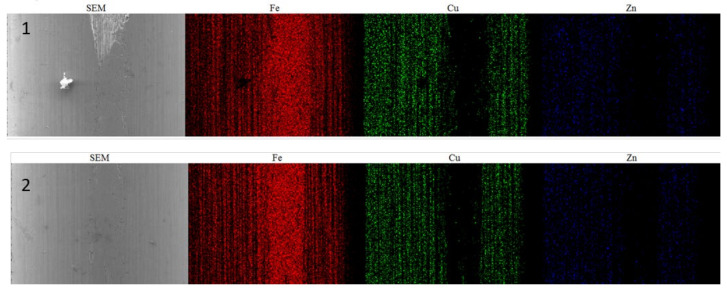
Mapping analysis of a sample containing defect.

The scratching process was performed during the drawing process, as confirmed by energy-dispersive spectroscopy analysis conducted both inside and outside of the defect location, as detailed in previous research [28]. This result was further validated through mapping analysis at the head of the defect (rectangle 1) and the surrounding area (rectangle 2), as shown in Figure 7. During the drawing process, the steel filament is deformed from a larger diameter to a thinner diameter through a system of dies. If the scratching occurs before this drawing process, there will be no significant difference between the inside and outside of the defect areas. Consequently, mapping analysis can be used to determine if there is any variation in the composition between the interior and exterior of the defect. This analysis helps to identify any differences in ingredients that may contribute to the defect, providing valuable insights into the root causes and potential solutions. Figure 8 illustrates the absence of copper (Cu) and zinc (Zn) in the vicinity of surface defects, consistent with the findings reported in [28]. This suggests that such failures occurred during the drawing process, as copper and zinc were coated on the steel wire surface prior to drawing. 

### 3.2. Measurement of Defect’s Depth

**Figure 9:**
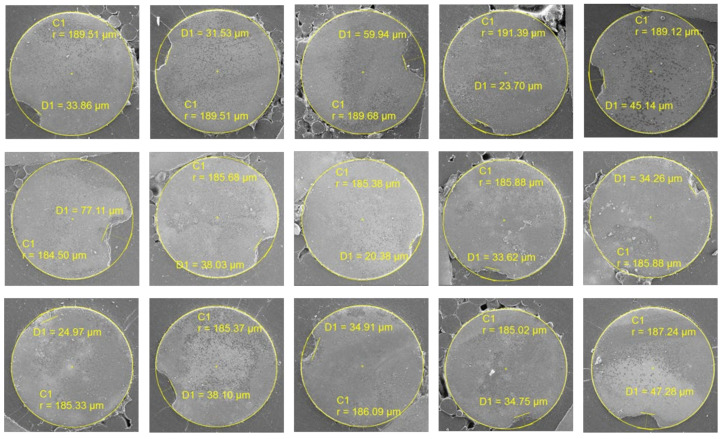
Cross-section of microdefects in Batch 1.

**Figure 10:**
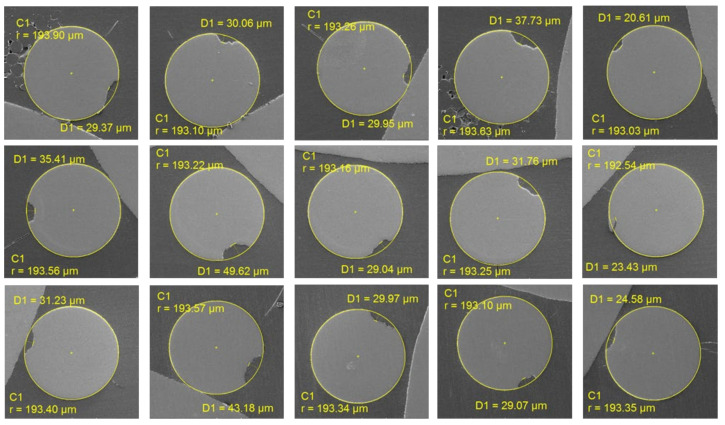
Cross-section of microdefects in Batch 2.

The defective samples were collected, and their cross-sections were captured using a scanning electron microscope. The depths of the cracks were measured, as presented in Table 2, and the cross-sections of the defective positions are shown in Figure 9 and Figure 10, corresponding to Batch 1 and Batch 2, respectively. The minimum and maximum measured depths are 20.38 µm and 77.11 µm, respectively, with an average defect depth of 35.07 µm. The measurement provides valuable insights into the quality and integrity of the steel filament. The data from Table 2 indicates variations in defect depths across two different batches, highlighting the complexity of defect depths and the challenges in achieving consistent quality of steel fibers.

### 3.3. Signal Amplitude and Defective Depth

**Figure 11:**
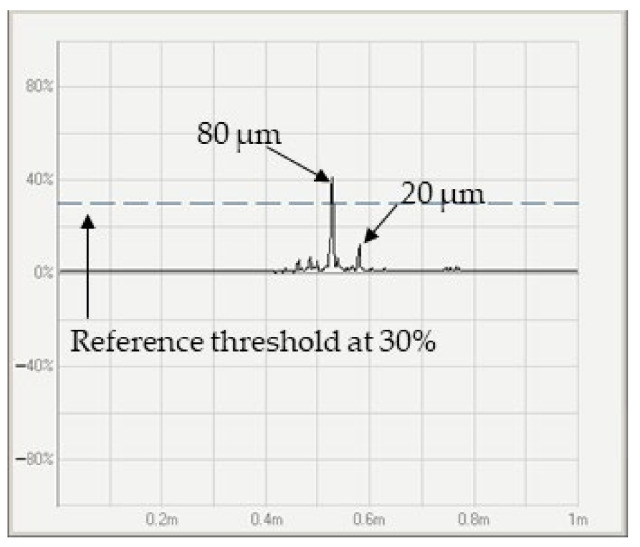
Amplitude signals of created defects of 20 and 80 µm.

**Figure 12:**
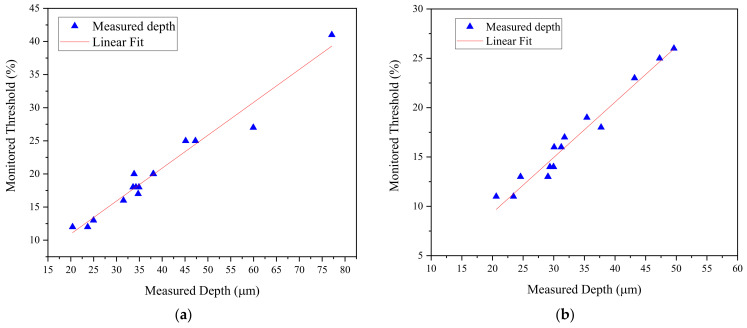
Depth and threshold of monitored defects: (**a**) Batch 1; (**b**) Batch 2.

Reference defects were created, as shown in Figure 3, and they were passed through the sensor to obtain the amplitude signals, which were used to establish the threshold for detecting a depth of 20 µm. As a result, the amplitude signals corresponding to the defects reached approximately 10% and 41% of the full-screen height (FSH) amplitude for depths of 20 and 80 µm, respectively. Additionally, noise was detected alongside the defects, with a level of 3% attributed to rust, as illustrated in Figure 11. When the sensor detected the defects with the desired threshold, the machine stopped the drawing process, allowing for the verification of monitored thresholds and phase angles on the monitor. Once verified, the drawing process resumed to complete the batch of experiments. Therefore, the threshold of eddy current sensing was set at 10%, corresponding to an expected depth of 20 µm, to identify defective specimens. Experimental depths were fitted with a linear function to calculate the threshold at 20 µm of measured depth. The relationship between the threshold and measured depth for the first and second batches of samples is depicted in Figure 12a and Figure 12b, respectively. The red line represents the linear fit between the monitored threshold and measured depth.

**Figure 13:**
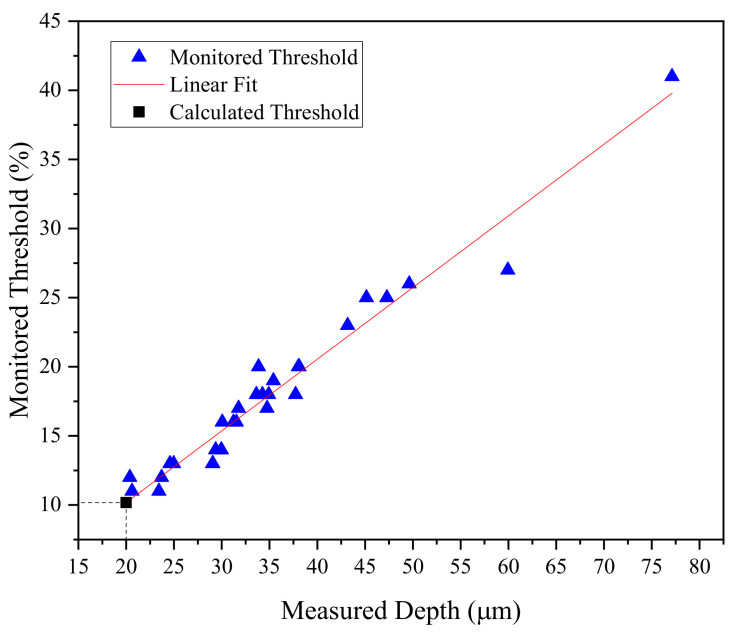
Threshold calculated for a depth of 20 μm.

When the data were combined, a linear function was fitted to calculate the threshold. As depicted in Figure 13, the depths of defects ranged from over 20 to 60 µm, with one sample having a depth exceeding 75 µm. The black square in Figure 13 represents the calculated threshold corresponding to 20 µm. The calculated threshold of 10.18% closely aligns with the set threshold of 10%, with a difference of only 1.77%.

### 3.4. Comparison Phase Angle Signal

**Figure 14:**
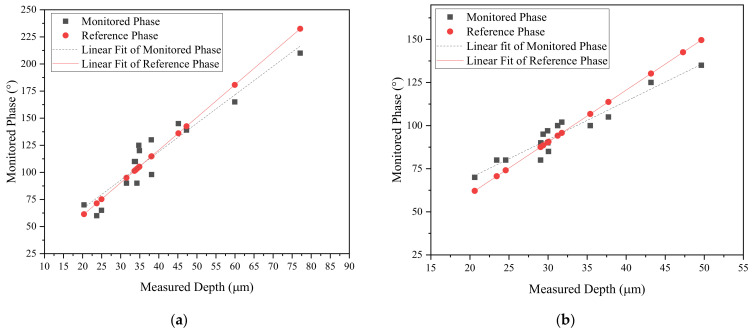
Depth and phase angle signal of monitored defects: (**a**) Batch 1; (**b**) Batch 2.

The relationship between the depth of the defect and the standard penetration depth is illustrated through Equations (1) and (2). This ratio establishes the defect’s position relative to the phase angle. The monitored phase angle is then compared to the reference phase angle, calculated from the ratio between the measured depth of defects and the standard penetration depth. This comparison is illustrated in Figure 14a and Figure 14b, corresponding to samples in Batch 1 and Batch 2, respectively.

**Figure 15:**
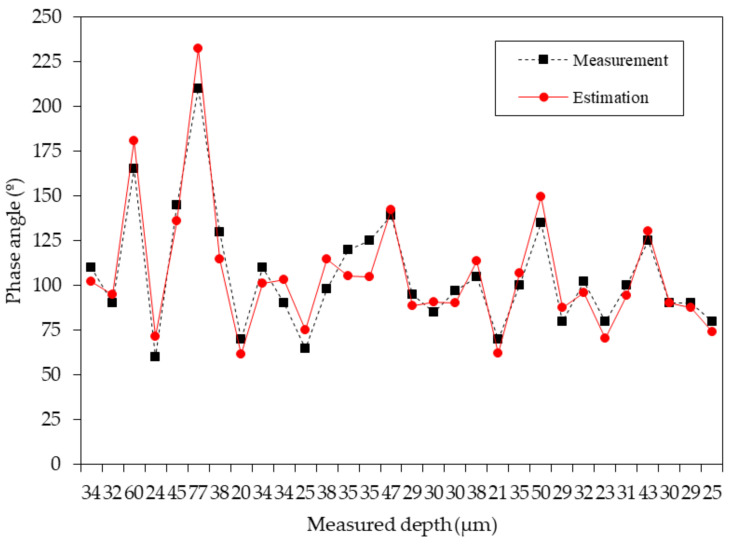
Comparison between measured phase signal and reference phase signal.

**Figure 16:**
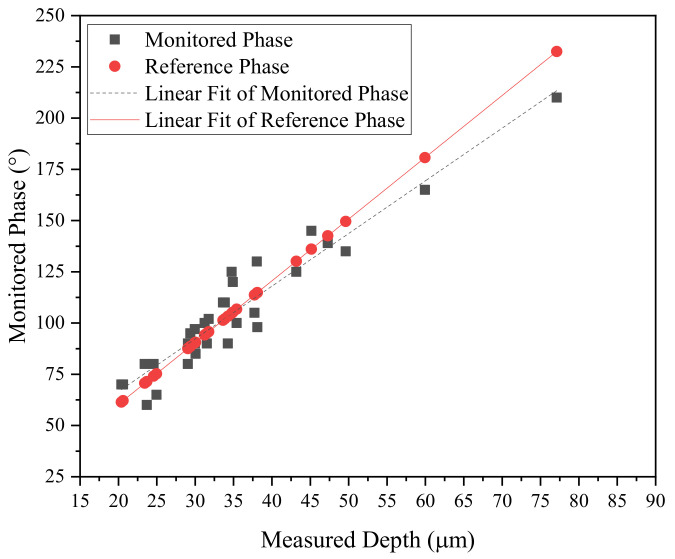
Comparison between measured phase signal and reference phase signal using RMSE.

The depth of microdefects and the detected phase angles underwent comparison through estimation, as illustrated in Figure 15. These results, amalgamated from two batches of analysis, formed the basis of comparison. However, for a comprehensive evaluation of the measured data versus the estimated results, a precise rearrangement of the dataset was required. Thus, the data were carefully reorganized to align with the sequence depicted in Figure 16, ensuring a coherent comparison between measurement and estimation outcomes. This process aimed to enhance the accuracy and reliability of the findings, then facilitating a deeper understanding of the studied phenomena.

A total of 30 defective samples were analyzed, and the phase angles between the experimental and theoretical results were compared. The red line represents the linear fit of the theoretical data, while the black line depicts the linear fit of the measured phase angles based on defective depth. The accuracy of this comparison was evaluated using the root mean square error (RMSE), which quantifies the difference between the measured phase angle and the reference angle. Figure 16 illustrates the comparison between the measured and reference phase angles for all samples. The resulting RMSE was found to be 10.53 degrees, equivalent to 3.49 µm. This discrepancy can be attributed mainly to the manual handling of depth measurement.

## 4. Conclusions

This paper presented several contributions that advance the understanding and detection of microdefects in steel filament manufacturing. Firstly, the method demonstrated high accuracy in identifying micro surface defects using eddy current sensing principles. By utilizing this technique, defects can be precisely located and analyzed, leading to improved quality control and product integrity. Secondly, the paper introduced an approach to verifying the setting conditions for defect detection by measuring defective depth relative to the standard penetration depth. This methodology provides a robust case study for non-destructive testing methods, offering insights into optimizing detection parameters for enhanced performance and reliability.

Moreover, the detailed image analysis of micro surface defects on steel filament contributed to the field of Failure Mode and Effect Analysis (FMEA). Understanding surface failures at the micro level is essential in cutting-edge manufacturing technologies, where even minor defects can have significant implications for product quality and performance.

However, the paper also highlighted challenges associated with manual handling activities, which can introduce discrepancies between theoretical and calculated phase angles. This underscores the need for advanced mechanisms that combine long-range movement and precision measurement capabilities to overcome such limitations and further enhance the accuracy and reliability of defect detection processes. In summary, this paper not only presents a methodology for defect detection and verification, but also establishes the critical role of microdefect analysis in advancing manufacturing quality and reliability.

## Figures and Tables

**Table 1:** Steel filament parameters.

Symbols	Unit	Definition	Value
*σ*	S/m	Electrical conductivity	$6.99\times 10^{6}$
*μ*	H/m	Magnetic permeability	$1.26\times 10^{-4}$
*ω*	Radian/second	Frequency	6,283,185

**Table 2:** Depths of microdefects.

Sample No.	Defect Depth Batch 1 (μm)	Defect Depth Batch 2 (μm)	Sample No.	Defect Depth Batch 1 (μm)	Defect Depth Batch 2 (μm)
1	33.86	29.37	9	33.62	31.76
2	31.53	30.06	10	34.26	23.43
3	59.94	29.95	11	24.97	31.23
4	23.70	37.73	12	38.10	43.18
5	45.14	20.61	13	34.91	29.97
6	77.11	35.41	14	34.75	29.07
7	38.03	49.62	15	47.28	24.58
8	20.38	29.04			

## Data Availability

Data are contained within the article.
